# Anti-CD19 CAR-T cell therapy in relapsed/refractory t(8;21) acute myeloid leukemia with aberrant CD19 expression

**DOI:** 10.3389/fimmu.2025.1617589

**Published:** 2025-07-21

**Authors:** Xiaomin Zhang, Lixin Wang, Jingqiao Qiao, Shuhong Wang, Lijun Wang, Lian Liu, Jiading Qin, Ziren Chen, Wenfa Huang, Yuanyuan Zheng, Huixin Peng, Junhui Mei, Hongxin Wang, Chuan Yu, Yisheng Li, Li Yu

**Affiliations:** ^1^ Department of Hematology and Oncology, Shenzhen University General Hospital, International Cancer Center, Hematology Institution, Haoshi Cell Therapy Institute of Shenzhen University, Shenzhen University Medical School, Shenzhen University, Shenzhen, China; ^2^ Guangdong Key Laboratory for Biomedical Measurements and Ultrasound Imaging, National-Regional Key Technology Engineering Laboratory for Medical Ultrasound, School of Biomedical Engineering, Shenzhen University Medical School, Shenzhen, China; ^3^ R&D Department, Shenzhen Haoshi Biotechnology Co., Ltd, Shenzhen, China

**Keywords:** t(8; 21) acute myeloid leukemia, aberrant CD19 expression, CD19 CAR-T cell therapy, hematological remission, molecular remission

## Abstract

**Background:**

T (8; 21) acute myeloid leukemia (AML) is a special type of acute leukemia, and exhibits a heterogeneous prognosis, with a long-term relapse rate of about 40%. Once t(8; 21) AML patients experience relapse, they have an extremely poor prognosis, with a 5-year overall survival rate of less than 15%. Therefore, it is crucial to develop effective strategies to improve the prognosis of relapsed/refractory (R/R) t(8; 21) AML. CD19 is a specific B-cell surface marker, but it is aberrantly expressed in 50-80 % of t(8; 21) AML patients. CAR-T cells targeting aberrant cell-surface antigens could induce the depletion of tumor cells without the destruction of hematopoiesis. Therefore, CD19 might be a promising target for CAR-T cell therapy in R/R t(8; 21) AML with aberrant CD19 expression. The present study is aimed to explore the efficacy and safety of CD19 CAR-T cell therapy in R/R t(8;21) AML with aberrant CD19 expression.

**Methods:**

In the present study, 3 R/R t(8;21) AML patients with aberrant CD19 expression were enrolled. After lymphodepleting chemotherapy, 3 patients received autologous CAR-T cell infusion at a dose of 1.0 × 10^6 cells/kg, 2.0 × 10^6 cells/kg, and 2.0 × 10^6 cells/kg, respectively.

**Results:**

They all achieved CD19 negativity approximately half a month after CD19 CAR-T cell infusion. These indicate CD19 CAR-T cell therapy is effective in R/R t(8;21) AML with aberrant CD19 expression. However, patient 1 and patient 2 rapidly relapsed within 3 months after CD19 CAR-T cell therapy. Subsequently, patient 1 received allogeneic hematopoietic stem cell transplantation (allo-HSCT). Fortunately, patient 1 achieved mCR 2 months after allo-HSCT.

**Conclusion:**

Considering the short-term remission of CD19 CAR-T cell therapy in R/R t(8;21) AML, allo-HSCT might be performed as soon as possible to consolidate the efficacy of CAR-T cell therapy and reduce the risk of relapse.

## 1 Introduction

Acute myeloid leukemia (AML) is the most common form of acute leukemia in adults. It has a poor prognosis, with a dismal 5-year survival rate of only 24%. T (8; 21) AML is a special type of acute leukemia with *RUNX1::RUNX1T1* fusion, which presents with favorable karyotype and accounts for 5% ~ 15% of AML (1). However, the prognosis of t (8; 21) AML is heterogeneous, and approximately 40% of t (8; 21) AML patients experienced relapse (2). Once t (8; 21) AML patients experience relapse, the prognosis of them is extremely poor, and the survival rate is usually less than 15%. It is well known that allogeneic hematopoietic stem cell transplantation (allo-HSCT) is the only potentially curative treatment option for relapsed/refractory (R/R) AML. However, approximately 40% of AML patients still experience relapse after allo-HSCT (3). Considering that the effective therapeutic options for R/R AML patients are currently limited, it is crucial to explore novel and effective therapeutic strategies to improve the prognosis of R/R AML.

Chimeric antigen receptor (CAR)-T cell therapy is a major breakthrough in cancer treatment, and it has achieved unprecedented responses in R/R B-cell malignancies in recent years, such as B-cell acute lymphoblastic leukemia (B-ALL), B-cell non-Hodgkin lymphoma (B-NHL), and multiple myeloma (MM) (4–6). However, due to antigen heterogeneity and the lack of specific target antigens, CAR-T cell therapy for R/R AML remains a major challenge. In particular, due to the overexpression of target antigens on hematopoietic stem cells (HSCs), such as CD123 and CD33, CAR-T cells targeting these co-expressed antigens on leukemic blast cells and HSCs may not only deplete malignant cells but also induce the clearance of bone marrow cells, resulting in severe myelosuppression (7, 8). This off-tumor on-target toxicity may be fatal and further limit the application of CAR-T cell therapy in R/R AML. In addition, CAR-T cell therapy targeting these co-expressed surface antigens are mostly at the preclinical stage.

At present, a number of studies have demonstrated that approximately 50-80% of t(8;21) AML patients present with aberrant expression of B-cell-specific surface marker CD19 on leukemic blast cells (9–13). Targeting aberrant CD19 expression could result in the depletion of these CD19-positive tumor cells without the destruction of hematopoiesis (14). Therefore, CD19 may also serve as a potential immuno-therapeutic target for AML with aberrant CD19 expression, such as t(8;21) AML and mixed-phenotype acute leukemia (14, 15). It is well-known that CD19 CAR-T cell therapy has achieved satisfactory efficacy in B-cell malignancies, including B-ALL and B-NHL. However, there is very little data on the application of CD19 CAR-T cell therapy in CD19-positive R/R AML (10). Considering the frequent expression of CD19 in t(8;21) AML patients, the present study is aimed to investigate the efficacy and safety of CD19 CAR-T cell therapy in R/R t(8;21) AML with aberrant CD19 expression.

## 2 Methods

### 2.1 Study approval and clinical protocols

Three R/R t(8;21) AML patients with CD19 aberrant expression were enrolled in the clinical trial of CD19 CAR-T cell therapy, which was approved by the Institutional Review Board at Shenzhen University General Hospital. Written informed consent was obtained from all the participants. Before CAR-T cell infusion, all patients received lymphodepleting chemotherapy with FC regimen (fludarabine 50 mg/m^2^ day -5 to day -3, cyclophosphamide 400 mg/m^2^ day -5 to day -3). Day 0 referred to the day on which CAR-T cells were infused.

### 2.2 CAR-T cell manufacturing

Peripheral blood mononuclear cells (PBMCs) were collected by apheresis and further isolated and purified by Ficoll density gradient centrifugation. Then T cells were isolated and activated by CD3/28 Dynabeads. After activation for 2 days, the lentiviral vector encoding CD19 CAR was applied for the T cells transduction. The CAR structure consists of a humanized anti- CD19 single-chain variable fragments (scfv), a CD8α hinge region, a 4-1BB co-stimulatory domain, and a CD3ζ signaling domain, as well as a truncated human epidermal growth factor receptor (tEGFR). The CAR-T cells were further expanded in X-vivo medium containing 10 ng/mL IL-7, 5 ng/mL IL-15, and 30 ng/mL IL-21 till the cell quantity reached the dose requirement.

### 2.3 Flow cytometry

To detect CD19-positive leukemic blasts, 8 mL bone marrow samples were collected in EDTA tubes at different time points. After red blood cell lysis, the remaining cells from each tube were stained with anti-human antibodies, including CD45-KO (Beckman Coulter, B36294), CD34-APC (BD biosciences, 652837), cMPO-FITC (BD biosciences,340580), CD11B-FITC (Beckman Coulter, IM0530), HLA-DR APC (BD Bioscience, 662909), CD64-PE (BD biosciences, 652830), CD14-APC (BD biosciences, 6657505), CD117-PE (BD Bioscience, 664936), CD33-PE-Cy5.5 (Beckman Coulter, B36289), CD13-PE-Cy7 (BD Bioscience, 662910), CD38-BV421(BD biosciences, 562444), CD19-PE-Cy7 (Beckman Coulter, IM3628), CD16-ECD (Beckman Coulter, B49216), and CD56-ECD (Beckman Coulter, B49214) for flow cytometric analysis. To detected the expansion of CAR-T cells which incorporate a truncated EGFR element, the following antibodies were used: EGFR-APC (Biolegend, 52906) and CD3-APC/Cyanine7 (Biolegend, 300426).

### 2.4 ELISA

Serum levels of IL-6, IL-8, and IL-10 were detected by commercial ELISA kits (R&D Systems, Minneapolis, USA) and the protocols were adopted according to manufacturer’s instructions.

## 3 Results

### 3.1 Patient characteristics

Three R/R t(8;21) AML patients were enrolled, and patient 3 had *c-KIT* mutation. The characteristics of the enrolled patients are summarized in **Table 1**. All the patients had been heavily pretreated, and the detailed information is available below.

**Table 1:** Baseline clinical characteristics of 3 R/R t(8;21) AML patients.

The characteristics of three patients			
Patients	Patient 1	Patient 2	Patient 3
Age	34	26	55
Sex (gender)	male	male	male
Diagnosis	t(8;21) AML (M2)	t(8;21) AML (M2)	t(8;21) AML (M2)
Karyotype	46, XY, t(8;21),(q22:;q22)	46, XY, t(8;21), (q22;q22), del(11)(q23)[19]/46, XY	46, XY, t(8;21),(q22;q22),
Gene mutation	ASXL1	c-KIT	ASXL1
EMD	no	no	cerebrospinal fluid
Allo-HSCT before CAR-T	no	no	no
CD19 expression levels on leukemic blasts (%)	100.00%	93.68%	76.44%

**Figure 1:**
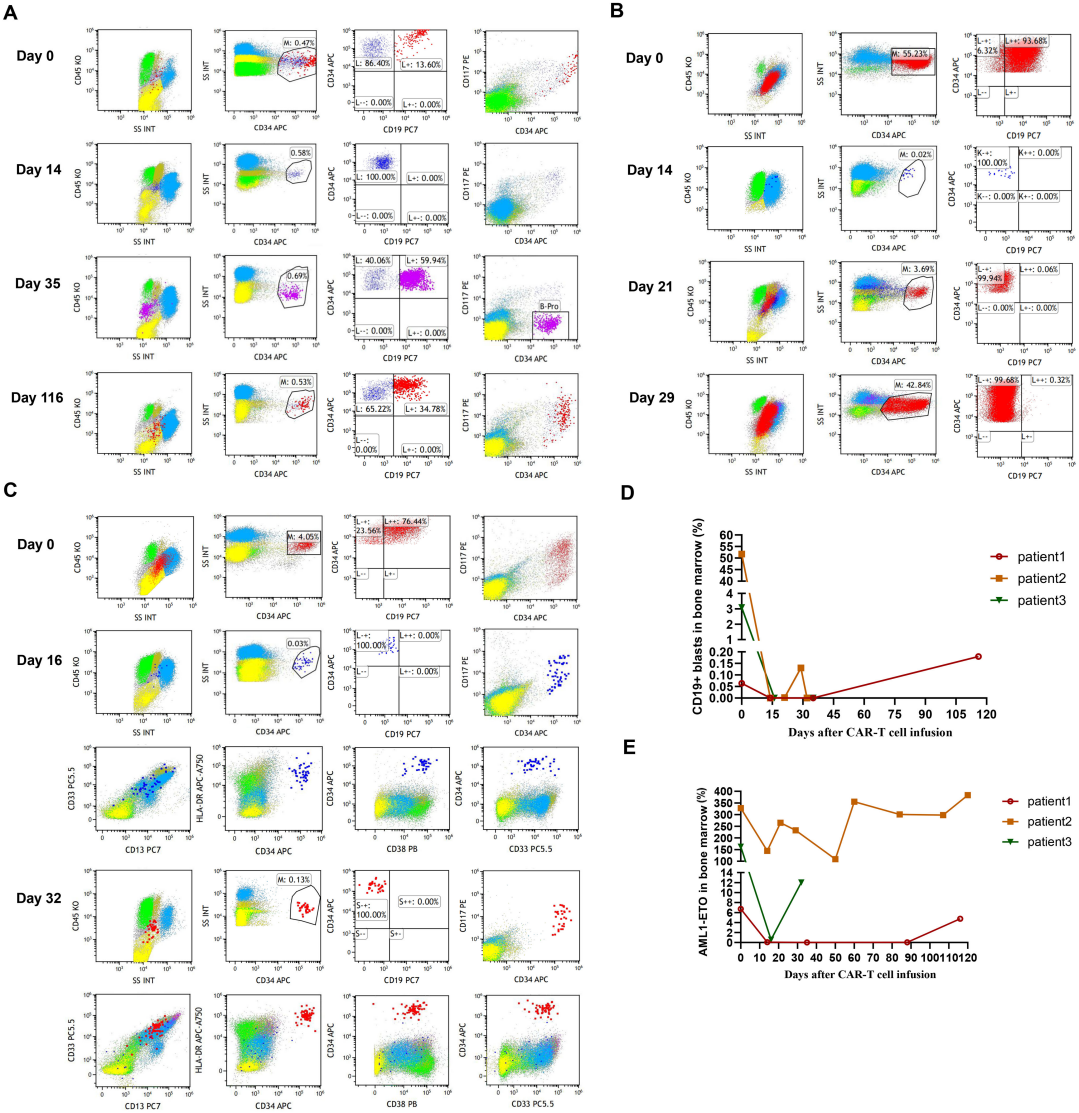
**(A)** The percentage of leukemic blasts in bone marrow after CAR-T cell infusion in patient 1. Red dots represent leukemic blasts and dark blue dots represent normal primitive hematopoietic cells, which are also applicable to patient 2 and patient 3. Purple dots represent B-progenitor cells. **(B)** The percentage of leukemic blasts in bone marrow after CAR-T cell infusion in patient 2. **(C)** The percentage of leukemic blasts in bone marrow after CAR-T cell infusion in patient 3. On day 16, no leukemic blasts were detected, as shown by the normal development and differentiation of primitive hematopoietic cells. On day 32, 0.13% of leukemic blasts were detected by FCM in bone marrow, which abnormally expressed several markers, such as CD38 dim, bright CD33, bright CD13, and bright HLA-DR. **(D)** The changes of CD19+ leukemic blasts in bone marrow after CAR-T cell infusion in 3 patients. **(E)** The transcript levels of *RUNX1::RUNX1T1* in bone marrow after CAR-T cell infusion in 3 patients.

Patient 1 is a 34-year-old man diagnosed with t(8;21) AML by cytogenetic and fluorescence-*in-situ* hybridization (FISH) studies in February 2023. Bone marrow smear and flow cytometry (FCM) showed more than 80.0% of leukemic blasts. His karyotype was 46, XY, t(8;21) (q22; q22). *RUNX1::RUNX1T1* rearrangement and *ASXL1* mutation were detected. The patient received standard “3 + 7” regimen with idarubicin and cytarabine (IA) as induction therapy, and then received three cycles of high dose cytarabine as consolidation therapy. After induction and consolidation therapy, he achieved molecular complete remission (mCR) with *RUNX1::RUNX1T1* gene copies and *ASXL1* mutation undetectable. Due to pulmonary infections, the patient didn’t receive allo-HSCT. In February 2024, FCM showed 55.33% blasts in peripheral blood, which indicated AML relapse. Then, the patient received two cycles of CDHAA regimen (Chidamide 30 mg/d d1 and d3; Decitabine 20 mg/d d1-5; Harringtonine 2mg/d d3-7; Cytarabine 100mg/d d3-7; Aclarubicin 20mg/d d3-7), and the patient achieved hematological complete remission (hCR) with 0.56% of *RUNX1::RUNX1T1 fusion* gene detectable. Due to minimal residual disease (MRD) positivity after multi-line of chemotherapy and the high levels of CD19 expression on leukemic blast cells (**Figure 1A**), the patient was enrolled in the clinical trial of CD19 CAR-T cell therapy to prevent hematological relapse. After lymphodepleting chemotherapy, he was treated with a single infusion of autologous CD19 CAR-T cells at a dose of 1.0 × 10^6 cells/kg on August 5, 2024.

Patient 2 is a 26-year-old man diagnosed with DLBCL in January 2022, and underwent 5 cycles of R-CHOP regimen as well as radiotherapy. Unfortunately, the patient failed to achieve remission and diagnosed with therapy-related t(8;21) AML with *c-KIT* mutation by cytogenetic and FISH studies in August 2024. His karyotype was 46, XY, t(8;21) (q22;q22), del(11) (q23). For the treatment of t(8;21) AML, the patient received IA regimen and CDHAA regimen. However, the patient failed to achieve hCR and experienced disease progression. Due to 55.23% of leukemic blasts and high levels of aberrant CD19 expression in bone marrow (**Figure 1B**), the patient was enrolled in the clinical trial of CD19 CAR-T cell therapy to deplete leukemic blasts. After lymphodepleting chemotherapy, he was infused with a single infusion of autologous CD19 CAR-T cells at a dose of 2.0 × 10^6 cells/kg on October 21, 2024.

Patient 3 is a 55-year-old man diagnosed with t(8;21) AML confirmed by cytogenetic and FISH studies in August 2017, and then received standard “3 + 7” IA regimen. After 2 cycles of IA regimen and 1 cycle of intermediate-dose cytarabine for consolidation therapy, the patient achieved mCR on December 2017. Due to the lack of suitable donors, the patient didn’t receive allo-HSCT. Unfortunately, 3.38% of *RUNX1::RUNX1T1* fusion gene was detected in December 2020, which suggested molecular relapse. Then, the patient underwent multi-line of chemotherapy, including cytarabine combined with etoposide, 4 cycles of DCAG (DAC 15 mg d1-5, Acla 20 mg d4-7, Ara-C 20 mg d1-7, G-CSF 300 μg d1-7), and 2 cycles of CDHAA regimen. After a variety of chemotherapy regimens, the patient still didn’t achieve molecular remission. Unfortunately, leukemic blasts were detectable in the cerebrospinal fluid, which indicated central nervous system leukemia (CNS-L). After intrathecal injection of methotrexate, cytarabine, and dexamethasone, CNS-L was controlled. Given MRD positivity and 76.44% of CD19 expression on leukemic blasts (**Figure 1C**), the patient was enrolled in the clinical trial of CD19 CAR-T cell therapy to prevent hematological and CNS relapse. After lymphodepleting chemotherapy, he was treated with a single infusion of autologous CD19 CAR-T cells at a dose of 2.0 × 10^6 cells/kg on October 21, 2024.

### 3.2 The kinetics of CD19 CAR-T cell expansion in R/R t(8;21) AML patients and objective responses

The efficacy of CD19 CAR-T cell therapy in R/R t(8;21) AML with CD19 aberrant expression was evaluated by bone marrow aspiration within half a month to 3 months.

**Figure 2:**
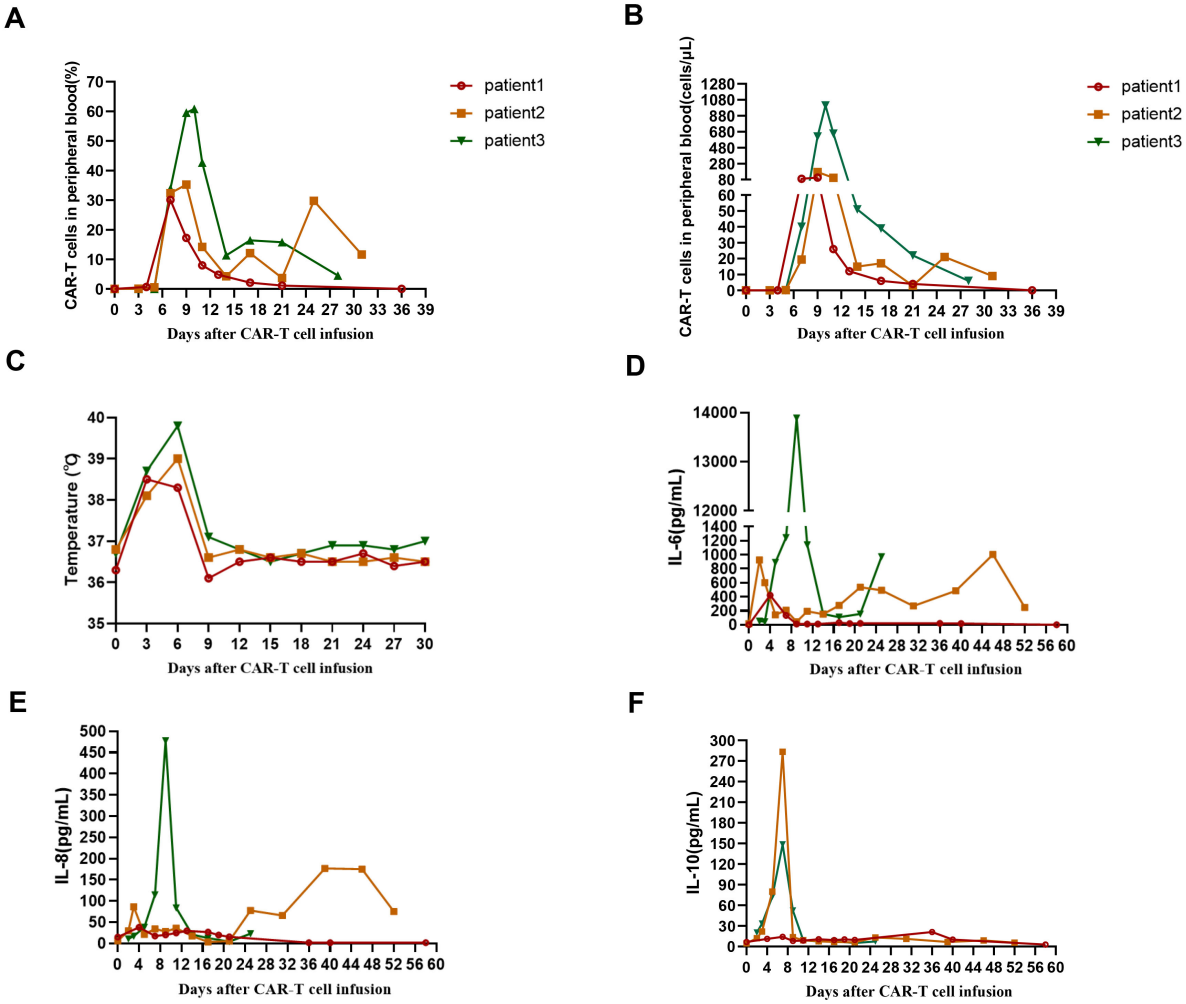
**(A)** The percentage of CD19 CAR-T cells in peripheral blood from 3 patients after CAR-T cell infusion. **(B)** The absolute number of CAR-T cells in peripheral blood from 3 patients after CAR-T cell infusion. **(C)** The changes of temperature in 3 patients after CAR-T cell infusion. **(D)** The levels of IL-6 in 3 patients after CAR-T cell infusion. **(E)** The levels of IL-8 in 3 patients after CAR-T cell infusion. **(F)** The levels of IL-10 in 3 patients after CAR-T cell infusion.

After lymphodepleting chemotherapy, patient 1 received autologous CD19 CAR-T cell infusion at a dose of 1.0 × 10^6 cells/kg on August 5, 2024. CAR-T cell expansion reached peak levels of 105 cells/μL in peripheral blood on day 9 as detected by FCM (**Figure 2B**). Surprisingly, leukemic blasts were completely depleted 14 days after CD19 CAR-T cell infusion (**Figures 1A, D**), and the levels of *RUNX1::RUNX1T1* fusion gene were decreased from 6.71% to 0.06% (**Figure 1E**). The patient achieved mCR with *RUNX1::RUNX1T1* fusion gene undetectable 35 days after CD19 CAR-T infusion (**Figure 1E**). Meanwhile, CD19 CAR-T cells were virtually undetectable 35 days after CD19 CAR-T infusion and B-progenitor cells were detected in bone marrow (**Figures 1A**, **2B**), which indicated CAR-T cell exhaustion. Unfortunately, 88 days after CD19 CAR-T cell therapy, 0.05% of *RUNX1::RUNX1T1* fusion gene was detected in bone marrow, which suggested molecular relapse in patient 1 (**Figure 1E**). Subsequently, CD19-positive leukemic blasts reemerged 116 days after CAR-T cell infusion (**Figures 1A, D**). Considering MRD positivity, the patient underwent matched sibling donor allo-HSCT on December 16, 2024. The total dose of CD34+ cells was 6.07 × 10^6 cells/kg. The patient achieved neutrophil and platelet engraftment 13 and 14 days after the infusion of allogeneic HSCs, respectively. On January 10, 2025, bone marrow chimerism testing by STR-PCR showed 99.85% donor chimerism. On January 23, 2025, 2.92% of leukemic blasts were detected in bone marrow by FCM, and the transcript levels of *RUNX1::RUNX1T1* fusion increased to 32.92%. Considering disease progression, cyclosporin A was discontinued. Subsequently, the patient developed grade III skin acute graft-versus-host disease (aGVHD) and grade II gut aGVHD, and presented with generalized rash and gastrointestinal symptoms, including abdominal pain, diarrhea, nausea, and vomiting. Fortunately, aGVHD was controlled by the combination treatment of cyclosporin A, mycophenolate mofetil, ruxolitinib, and methylprednisolone. On February 12, 2025, leukemic blasts and *RUNX1::RUNX1T1* fusion gene were not detected in bone marrow, and bone marrow chimerism testing by STR-PCR showed 100% donor chimerism. We speculate that leukemic blasts were depleted by graft versus leukemia effects. So far, patient 1 remained in mCR.

After lymphodepleting chemotherapy, patient 2 received autologous CD19 CAR-T cell infusion at a dose of 2.0 × 10^6 cells/kg on October 21, 2024. CAR-T cell expansion reached peak levels of 173 cells/μL in peripheral blood on day 9 as detected by FCM (**Figure 2B**). Surprisingly, leukemic blasts were completely depleted 14 days after CD19 CAR-T cell infusion, including CD19-positive tumor cells (**Figures 1B, D**), and the levels of *RUNX1::RUNX1T1* fusion gene decreased from 327.64% to 144.82% (**Table 2**, **Figure 1E**). These suggested that patient 2 achieved hCR14 days after CD19 CAR-T cell therapy. Unfortunately, 3.69% of leukemic blasts were detected in bone marrow by FCM 21 days after CD19 CAR-T cell therapy (**Figure 1B**), and the levels of leukemic blasts remarkably increased to 42.84% on 29 day (**Figure 1B**), which indicated the patient rapidly experienced hematological relapse.

**Table 2:** Response of CD19 CAR-T cell therapy in 3 R/R t(8;21) AML patients.

Response of CD19 CAR-T cell therapy			
Patients	Patient1	Patient2	Patient3
The time of the complete depletion of leukemic blasts detected by FCM	14 days	14 days	16 days
Best response	mCR	hCR	FCM MRD-
Best response time	35 days	14 days	16 days
CD19 expression at best response	0	0	0
Decrease of *RUNX1::RUNX1T1* levels from CAR-T cell infusion to best response	from 6.71% to 0%	from 327.64% to 144.82%	from 164.40% to 0.58%
The duration of best response	53 days	15 days	16 days
Relapse or FCM MRD+	molecular relapse	hematological relapse	FCM MRD+
CD19 expression levels on leukemic blasts after relapse or FCM MRD+	100%	0.32%	0%

After lymphodepleting chemotherapy, patient 3 was also treated with a single infusion of autologous CD19 CAR-T cells at a dose of 2.0 × 10^6 cells/kg on October 21, 2024. CAR-T cell expansion reached peak levels of 1009 cells/μL in peripheral blood on day 10 (**Figure 2B**). Sixteen days after CD19 CAR-T cell infusion, flow cytometric analysis showed that CD19-positive leukemic blasts were completely depleted (**Figures 1C, D**), and the copy numbers of *RUNX1::RUNX1T1* fusion gene decreased from 187233 copies/μg to 1908 copies/μg, and the relative values of *RUNX1::RUNX1T1* fusion gene also decreased from 164.40% to 0.58% (**Table 2**, **Figure 1E**). However, 32 days after CD19 CAR-T cell therapy, 0.13% of leukemic blasts were detected in bone marrow by FCM and showed CD19-negative expression (**Figure 1C**), and the copy number of *RUNX1::RUNX1T1* fusion gene in bone marrow was 19024 copies/μg, and the relative values of *RUNX1::RUNX1T1* fusion gene was 12.03% (**Figure 1E**). Ultimately, he died of septic shock 49 days after CD19 CAR-T cell infusion.

### 3.3 Adverse effects of CD19 CAR-T cell therapy in 3 R/R t(8;21) AML patients

Cytokine release syndrome (CRS) occurred in all 3 patients. Patient 1 experienced grade 3 CRS (**Table 3**), mainly manifested as fever and hypotension, which was controlled by intravenous rehydration, tocilizumab, norepinephrine, and dopamine. Patient 2 and patient 3 experienced grade 2 and grade 1 CRS, respectively (**Table 3**). Especially, patient 3 presented with persistent high fever for several days, and the peak levels of IL-6 reached 13890.20 pg/mL on day 9 (**Figure 2D**, **Table 3**). To alleviate the clinical symptoms of CRS, patient 2 and patient 3 were also treated with tocilizumab. Due to agranulocytosis mediated by long-term chemotherapy as well as CAR-T cell therapy, all 3 patients received prophylactic anti-infective treatment, including acyclovir, voriconazole, and imipenem, and patient 3 received granulocyte colony-stimulating factor. Unfortunately, patient 3 still suffered from multiple severe infections within 49 days after CD19 CAR-T cell infusion, mainly manifested as bacterial infections, including Klebsiella pneumoniae and Pseudomonas aeruginosa. Eventually, patient 3 died of septic shock 49 days after CD19 CAR-T cell infusion, despite aggressive anti-infective treatment, such as a combination of meropenem and tigecycline. Immune effector cell-associated neurotoxicity syndrome (ICANS) was not observed in all 3 patients. Furthermore, due to thrombocytopenia and severe anemia, patient 2 and patient 3 received multiple platelet and leukocyte-depleted red blood cell transfusions within 1 month after CAR-T cell infusion.

**Table 3:** Toxicities of CD19 CAR-T cell therapy in 3 R/R t(8;21) AML patients.

Toxicities of CD19 CAR-T cell therapy			
Patients	Patient 1	Patient 2	Patient 3
CRS grade	3	2	1
Tocilizumab Tx/N doses	+ 8 mg/kg/2 doses	+8 mg/kg/2 doses	+ 8 mg/kg/1 doses
Maximal IL-6 (mg/L)	422.1	923.8	13890.2
The peak levels of CAR-T cells	105 cell/μL	173 cell/μL	1009 cell/μL
ICANS	no	no	no
Other adverse effects	no	no	severe infections

## 4 Discussion

R/R AML has a poor prognosis with a 5-year survival rate of about 24%. Up to now, the therapeutic options for R/R AML are limited. Allo-HSCT is the only curative treatment option, but approximately 40% of AML patients still relapse after allo-HSCT. Therefore, it is crucial to explore effective therapeutic strategies to improve the prognosis of R/R AML. In recent years, CAR-T cell therapy has revolutionized the outcomes of R/R B-cell malignancies and shown great promise in the treatment of R/R AML. However, there are various difficulties for CAR-T therapy in R/R AML, including the lack of specific target antigens and off-target toxicities. In particular, due to the co-expression of pan-myeloid markers on leukemic blast cells and hematopoietic cells, such as CD33, CD123, CLL-1, CD70, and CD44v6, CAR-T cells targeting these antigens may result in a higher risk of the destruction of hematopoiesis (7, 16, 17). The prolonged myeloablation after CAR-T cell therapy in AML is fatal, which is mainly manifested as neutropenic infections and bleeding (16). Thereby, identification of novel antigens which are expressed on leukemic blasts but not normal hematopoietic cells may help to improve the safety of CAR-T cell therapy in R/R AML.

Aberrant antigen expression is a hallmark of AML. Aberrantly expressed antigens are absent from normal hematopoietic stem and progenitor cells, so targeting these surface antigens may result in the depletion of tumor cells without myelotoxicity. It has been demonstrated that more than 50% of t(8;21) AML patients present with aberrant CD19 expression on leukemic blasts (13), which indicates that CD19 is a promising target for CAR-T cell therapy in CD19-positive t(8;21) AML patients. However, there is very little data on the application of CD19 CAR-T cell therapy in R/R t(8;21) AML. Therefore, the present study investigated the efficacy and safety of CD19 CAR-T cell therapy in R/R t(8;21) AML with aberrant CD19 expression. In the present study, flow cytometric analysis showed leukemic blasts were rapidly depleted in all three patients within 16 days after CAR-T cell infusion (**Table 2**, **Figures 1A–C**). Patient 1 achieved mCR with *RUNX1::RUNX1T1* fusion gene undetectable 35 days after CD19 CAR-T cell therapy (**Table 2**, **Figure 1E**), and patient 2 achieved hCR 14 days after CD19 CAR-T cell infusion (**Table 2**, **Figures 1B, E**). Flow cytometric analysis showed leukemic blasts were completely depleted 16 days after CD19 CAR-T cell infusion in patient 3 (**Table 2**, **Figure 1C**), and the levels of *RUNX1::RUNX1T1* fusion gene rapidly decreased from 164.40% to 0.58% (**Table 2**, **Figure 1E**). These demonstrated that CD19 CAR-T cell therapy was effective in R/R t(8;21) AML patients with aberrant CD19 expression, which might further expand the application of CD19 CAR-T cell therapy, beyond B-cell malignancies. However, patient 1 and patient 2 rapidly relapsed after CD19 CAR-T cell therapy in the present study, especially patient 2. Patient 1 experienced molecular relapse 88 days after CD19 CAR-T cell therapy, with MRD positivity and CD19-positive expression detected by FCM 116 days after CAR-T cell infusion. These may be partially attributed to CD19 CAR-T cell exhaustion (**Figures 2A, B**), which was consistent with the regeneration of B-progenitor cells detected by FCM 35 days after CAR-T cell infusion (**Figure 1A**). Patient 2 experienced hematological relapse 29 days after CAR-T cell infusion, and the vast majority of leukemic blasts were CD19-negative (**Figure 1B**). Similarly, 0.13% of leukemic blasts were detected in bone marrow by FCM 32 days after CAR-T cell infusion in patient 3, manifested by CD19 negativity, and the transcript levels of *RUNX1::RUNX1T1* in bone marrow increased to 12.03%. Unfortunately, patient 3 died of septic shock 49 days after CD19 CAR-T cell infusion, which may be associated with the impaired immune function mediated by previous long-term chemotherapy and CD19 CAR-T cell therapy (18).

It is well known that there are several leading causes contributing to tumor recurrence after CAR-T cell therapy, including antigen escape and CAR-T cell exhaustion (19–21), which were also observed in our study. On the one hand, the potent selective pressure of CD19 CAR-T cell therapy may trigger the mutation or loss of the target antigen CD19, also known as CD19 antigen escape (19, 20, 22). In addition, CD19 CAR-T cells may not eradicate all tumor cells due to the pre-existing CD19-negative leukemic subclones. These CD19-negative leukemic subclones may also become predominant subsets under the potent selective pressure of CD19 CAR-T cell therapy (23), as seen in patient 2 and patient 3, which might eventually result in CD19-negative relapse. CD19-negative relapse after CD19 CAR-T cell therapy in R/R t(8;21) AML was also observed in the previous study (10). On the other hand, CD19 CAR-T cell exhaustion could result in CD19-positive relapse, as seen in patient 1. Regardless, CD19 CAR-T cells rapidly reduced tumor burden in R/R t(8;21) AML patients with aberrant CD19 expression in our study. Therefore, CD19 CAR-T cell therapy may be serve as a promising bridging therapy prior to allo-HSCT in CD19-positive R/R t(8;21) AML patients. Furthermore, considering that the duration of remission induced by CD19 CAR-T cell therapy is short in the present study, allo-HSCT should be performed as soon as possible to eradicate the residual tumor cells, including CD19-negative subclones, and then reduce the risk of relapse and achieve long-term tumor remission in R/R t(8;21) AML (10, 22).

In addition, patient 2 is a CD19-positive R/R t(8;21) AML patient with *c-KIT D816V* mutation, which is recognized to an independent adverse prognostic factor in t(8; 21) AML and could significantly increase the risk of tumor recurrence (24). Several studies have confirmed that CAR-T cell therapy is able to overcome the unfavorable prognosis in high-risk lymphoma and multiple myeloma, such as double hit or double expressor lymphomas, and multiple myeloma with extramedullary disease or high-risk cytogenetics (25–27). In the present study, it seems that CAR-T cell therapy is unlikely to overcome the poor prognosis of *c-KIT* mutation in R/R t(8;21) AML. At present, it has been demonstrated that avapritinib could specifically target *c-KIT* mutation and induce rapid and deep remission in t(8; 21) AML (28–30). Unfortunately, avapritinib was ineffective in patient 2. Subsequently, he will receive allo-HSCT as salvage therapy.

## 5 Conclusion

In conclusion, CD19 CAR-T cell therapy for R/R t(8;21) AML with CD19 aberrant expression is effective and safe, which broadens the application of CD19 CAR-T cell therapy in hematological malignancies, beyond B-cell malignancies. It provides a new strategy for the treatment of R/R t(8;21) AML. Although CD19 CAR- T cell therapy could reduce CD19-positive tumor burden in R/R t(8;21) AML patients with CD19 aberrant expression, tumor relapses occur rapidly due to CAR-T cell exhaustion or antigen escape. Therefore, allo-HSCT should be performed as soon as possible to consolidate the efficacy of CD19 CAR-T cell therapy and eradicate the residual tumor cells. CD19 CAR-T cell therapy combined with allo-HSCT may be an alternative strategy to overcome the heterogeneity of R/R t(8; 21) AML with CD19 aberrant expression. In addition, due to the limited number of patients in the present study, large-scale clinical trials are required to confirm the efficacy of CD19 CAR T- cell therapy as well as the combination of CD19 CAR T- cell therapy and allo-HSCT in CD19-positive R/R t(8;21) AML.

## Data Availability

The original contributions presented in the study are included in the article/supplementary material. Further inquiries can be directed to the corresponding authors.
